# Antivirus applied to JAR malware detection based on runtime behaviors

**DOI:** 10.1038/s41598-022-05921-5

**Published:** 2022-02-04

**Authors:** Ricardo P. Pinheiro, Sidney M. L. Lima, Danilo M. Souza, Sthéfano H. M. T. Silva, Petrônio G. Lopes, Rafael D. T. de Lima, Jemerson R. de Oliveira, Thyago de A. Monteiro, Sérgio M. M. Fernandes, Edison de Q. Albuquerque, Washington W. A. da Silva, Wellington P. dos Santos

**Affiliations:** 1 Department of Computing, University of Pernambuco, Recife, Brazil; 2 Electronics and Systems Department, Federal University of Pernambuco, Recife, Brazil; 3 Biomedical Engineering Department, Federal University of Pernambuco, Recife, Brazil

**Keywords:** Engineering, Electrical and electronic engineering

## Abstract

Java vulnerabilities correspond to 91% of all exploits observed on the worldwide web. The present work aims to create antivirus software with machine learning and artificial intelligence and master in Java malware detection. Within the proposed methodology, the suspected JAR sample is executed to intentionally infect the Windows OS monitored in a controlled environment. In all, our antivirus monitors and considers, statistically, 6824 actions that the suspected JAR file can perform when executed. Our antivirus achieved an average performance of 91.58% in the distinction between benign and malware JAR files. Different initial conditions, learning functions and architectures of our antivirus are investigated. The limitations of commercial antiviruses can be supplied by intelligent antiviruses. Instead of blacklist-based models, our antivirus allows JAR malware detection preventively and not reactively as Oracle’s Java and traditional antivirus modus operandi.

## Introduction

Currently, through the use of social network applications, global computer networks have spread widely, and their applications are concerned not only with entertainment and leisure but also with work. A large portion of the content provided by the global computer network is developed using Java technology. In the United States, Java applications are present in 97% and 89% of company computers and personal computers, respectively^1^. From servers to personal computers to mobile and dedicated devices, Java applications have been used in various computers^2^.

The goal of Java technology is to focus on creating applications that can be executed on various OSs (operating systems) and computer-controlled devices. Unlike machine language instructions, Java applications are OS-independent. Additionally, Java applications are portable, so they can be executed in any OS that includes a JVM (Java Virtual Machine) without modification or an extension. It should be emphasized that the JVM has been implemented in thousands of OSs. The conclusion is that Java applications have good portability. They can work on approximately 3 billion devices, including servers, personal computers, printers, smartphones, tablets, smart credit cards, and household appliances^3^.

Contrary to its popularity, incidents of targeted attacks using Java-based malware has been on the rise in recent years^4,5^; between 2012 and 2013, the number of vulnerabilities exploiting Java increased by more than 300%^5^. Java vulnerabilities usually target personal computers, technically named endpoints. Then, once on an endpoint, Java malware maliciously exploits daily activities, such as navigating the internet or using utilities. Therefore, bank passwords, social networks, photos or private videos may be stolen due to cyber infections. Java vulnerabilities correspond to 91% of all monitored exploits worldwide. Microsoft Word, Adobe Reader, Microsoft Excel and Microsoft PowerPoint correspond to 3%, 3%, 2% and 1% of all monitored exploits^4^.

One of the reasons for the failure of the Java security manager is the decrease in the catalog of new virtual viruses by its manufacturer. The modus operandi of the Java security manager is to identify malicious files based on signatures. Similar to Oracle Java, commercial antivirus software can also identify malicious files with signature features. Thus, this paper proposes to study (i) 86 kinds of commercial antivirus software to identify Java malware files, especially JAR (Java ARchive) files. The JAR malware detection range is from 0 to 99.10%, depending on the antivirus software. On average, 34.95% of virtual viruses were detected.

In our paper, approximately 31.39% of antivirus software could not diagnose any kind of malicious sample. It is worth noting that in our research, the malicious behavior of the analyzed JAR malware was recorded by incident responders. Even so, more than one-third of evaluated commercial antivirus software is not aware of the existence of the JAR malware file under investigation. Note the impediment of conventional antiviruses for the vigor of large-scale and real-time administrations.

The state-of-the-art antivirus recommends preventively extracting features of suspicious files before execution^6^. The executable file is submitted to the reverse engineering process. Then, the assembly code associated with the executable file can be studied so that the malicious intent of the suspicious file can be investigated. The features from the assembly code are used as input attributes to the artificial neural network used as the classifier. Neural network-based antiviruses accomplish an average performance of more than 90% in distinguishing benign and malware samples^6^.

As a side effect, the static analysis presents severe deficiencies when submitted to obfuscated malware, although several sophisticated static analysis techniques have been developed that show promising results^7^. As a digital antiforensics strategy, malware employs runtime code packaging and obfuscation. Therefore, the original instructions, made by the server, are different from those executed at runtime on the personal computer^7^. It is concluded that static feature research can be easily bypassed by obfuscation strategies^7^.

The static feature methodology constraint to precisely identify new types of malicious applications moved the focal point of malware research to feature exploration that can recognize malicious behavior as a process, named the dynamic approach, instead of a static approach. Then, our authorial antivirus performs the dynamic analysis of Java malware. Our feature extraction concerns the traces of calls made by all processes generated by the malware, files being created, deleted and downloaded by the malware while it is running, memory dumps of the malware processes, and network traffic tracking in PCAP format. The main advantage of the dynamic approach is that behavioral features are insensitive to low-level (bytecode) mutation techniques, such as runtime packing or obfuscation^7^. Overall, our dynamic feature extraction monitors 6,824 behaviors that the suspected JAR file can make when executed.

Then, the malicious behaviors originating from the suspect files serve as input attributes to neural networks. In this work, we employ an mELM (morphological ELM) neural network, an ELM with a hidden-layer kernel, which is inspired by erosion and dilation image processing morphological operators. The proposed paper claims that the morphological kernel can fit any critical decision. Mathematical morphology refers to studying the shape of objects in an image by using the mathematical theory of intersection and union of the set^8^. Then, the morphological operation naturally processes the shape detection of the object present in the image^8^. By interpreting the boundary decision of the neural network as an n-dimensional image, where n is the number of extracted features, our morphological ELM can naturally detect and model the mapping to different types of *n*-dimensional regions in any machine learning. Authorial antiviruses achieve an average performance of 91.58% in distinguishing between benign and malware Java applications accompanied by an average training time of 52.36 seconds.

This work is organized as follows. In Sect. 2, we present the limitations of commercial antiviruses. In Sect. 3, we discuss the state of the art regarding artificial intelligence antiviruses. In Sect. 4, we present the proposed methodology. In Sect. 5, we make a comparison between the authorial ELM network and classic ELM networks. In Sect. 6, we show the results and some discussions. Finally, in Sect. 7, we present the general conclusions and discuss the perspectives of our work.

## Commercial antiviruses limitation

Although it has been questioned for more than a decade, the modus operandi of antiviruses is based on signatures when the suspect file is consulted on datasets named in a blacklist. Therefore, it is enough that the hash of the investigated file not be in the antivirus blacklist to malware not to be detected. The hash functions are a unique identifier for a given file.

Given the limitations of commercial antiviruses, it is not a difficult task to develop and distribute variants of malicious applications. To do this, it is enough to make small alterations in the original malware with routines that, effectively, do not have any usefulness, such as repetition loops and conditional branches without instructions in their scopes. These alterations without usefulness, however, turn the hash of the modified malware from the hash of the original malware. Consequently, malware augmented with null routines is not recognized by the antivirus that recognizes the initial malware. It should be emphasized that the existence of exploits responsible for creating and distributing, in automated form, variants of the same original malware. It is concluded that antiviruses, based on signatures, have null effectiveness when submitted to variants of the same software^9,10^.

Through the VirusTotal platform, this proposed work explores 86 commercial antiviruses with their respective results presented in Table 1. We utilized 998 malicious JARs obtained from the REJAFADA dataset^11^. The objective of the work is to verify the number of malicious samples cataloged by antiviruses. The motivation is that the acquisition of new malware is primordial to combat malicious activities.

The larger the dataset, named the blacklist, the better it tends to be the defense given by the antivirus. First, JAR malware is sent to the server belonging to the VirusTotal platform. At this point, the JAR files were analyzed by VirusTotal’s 86 commercial antiviruses. Then, the antiviruses give their diagnostics for JAR samples submitted to the server. VirusTotal allows three different types of diagnostics to be issued: benign, malware, and omission.

For the first VirusTotal possibility, the antivirus detects the maliciousness of the suspicious file. Within the proposed experimental environment, all submitted samples are malware documented by incident responders. The antivirus hits when it recognizes the malignancy of the investigated file. Malware detection shows that the antivirus offers robust support against digital invasions. In the second possibility, the antivirus certifies the benignity of the defined file. Then, in the proposed study, when the antivirus claims the file is benign, it is a false negative, as all the samples sent are malicious. In other words, the investigated file is malware; however, the antivirus mistakenly attests to it being benign. Within the third possibility, the antivirus does not give an analysis of the suspect application. The omission shows that the investigated file was never evaluated by the antivirus, so cannot be evaluated in real time. The omission of diagnosis by antivirus points to its limitation on large-scale services.

Table 1 shows the results of the 86 antivirus products evaluated. The McAfee-GW-Edition antivirus achieved the best performance by detecting 99.10% of the investigated malware. One of the largest adversities in combining malicious applications is the fact that antivirus manufacturers do not share their malware blacklists due to commercial disputes. Through the analysis in Table 1, the proposed work points to an aggravating factor of this advantage: the same antivirus manufacturer does not share their databases among their different antiviruses. Observe, for example, that McAfee-GW-Edition and McAfee antiviruses belong to the same company. Their blacklists, although robust, are not shared between themselves. Therefore, the commercial strategies of the same company disturb the confrontation against malware, which demonstrates that antiviral manufacturers are not necessarily concerned with avoiding cyber invasions but with optimizing their business incomes.

Malware identification ranged from 0 to 99.10%, depending on the antivirus. Overall, the 86 antiviruses identified 34.95% of the examined malware, with a standard deviation of 40.92%. The elevated standard deviation shows that recognizing malicious files can change abruptly depending on the chosen antivirus. The protection against digital intrusions is in the function of choosing a vigorous antivirus with an expansive and upgraded blacklist. Overall, antiviruses certified false negatives in 33.90% of the cases, with a standard deviation of 40.45%. Attention to the benignity of malware can be implicated in unrecoverable damages. A person or institution, for instance, may begin to trust a certain malicious application when, in fact, it is malware. Nevertheless, as an unfavorable aspect, approximately 31.39% of antiviruses did not express an opinion on any of the 998 malicious samples. On average, the antiviruses were omitted in 31.15% of the cases, with a standard deviation of 45.61%. The omission of the diagnosis focuses on the constraint of antivirus in recognizing malware in real time.

Due to difficulty in combating malicious applications, commercial antiviruses do not have a pattern in the classification of malware, as found in Table 2. We have chosen 3 of the 998 JAR malware to exemplify the miscellaneous classifications given by commercial antiviral activities. As there is no pattern, the antiviruses use the names that they want; for example, a company can identify JAR malware as “Android: RuFraud-I” and a second company can identify it as “Artemis! 9EF6966B98A5;”. Therefore, the lack of a pattern disturbs the cybersecurity strategies since each category of malware must have different treatments (vaccines). It is concluded that it is impracticable for supervised machine learning to adopt pattern recognition for JAR malware categories. Due to this confusing tangle of multiclass classification provided by specialists (antiviruses), as seen in Table 2, it is statistically improbable that any machine learning technique will acquire generalization capability.

**Table 1:** Results of commercial antiviruses. Expanded results of 86 worldwide commercial antiviruses are in the authorial repository^11^.

Antivirus	Detection (%)	False negative (%)	Omission (%)
McAfee-GW-Edition	99.10	0.90	0.00
NANO-Antivirus	97.70	2.20	0.10
AegisLab	97.60	2.10	0.30
Kaspersky	96.80	2.90	0.30
ZoneAlarm	96.70	2.90	0.40
Avast	96.60	3.30	0.10
AVG	96.60	3.30	0.10
ESET-NOD32	95.90	4.10	0.00
McAfee	95.60	4.40	0.00
Avira	94.80	3.30	1.90
Cylance	0.20	0.00	99.80
WhiteArmor	0.20	91.30	8.50
Alibaba	0.20	98.50	1.30
ALYac	0.10	95.80	4.10
Bkav	0.10	97.60	2.30
Paloalto	0.00	0.00	100.00
SentinelOne	0.00	0.00	100.00
Endgame	0.00	0.00	100.00
CrowdStrike	0.00	0.00	100.00
Agnitum	0.00	0.00	100.00

**Table 2:** Result of the submission of three malware to VirusTotal. Expanded results of 86 worldwide commercial antiviruses are in the authorial repository^11^.

Antivirus	VirusShare$_A$	VirusShare$_B$	VirusShare$_C$
McAfee-GW-Edition	Artemis!Trojan	Artemis	PWS-Zbot.gen.jr
NANO-Antivirus	Trojan.Android.SMSSend.numyx	Trojan.Android.Opfake.oefcg	Trojan.Java.CVE20113544.cspflc
AegisLab	Troj.Sms.Androidos!c	SUSPICIOUS	Troj.W32.Generic!c
Kaspersky	HEUR:Trojan-SMS. AndroidOS.Fakelogo.a	HEUR:Trojan-SMS. AndroidOS.Fakelogo.a	HEUR:Trojan. Win32.Generic
ZoneAlarm	HEUR:Trojan-SMS. AndroidOS.Fakelogo.a	HEUR:Trojan-SMS. AndroidOS.Fakelogo.a	HEUR:Trojan. Win32.Generic
Avast	Android:RuFraud-I	Android:RuFraud-I	Java:CVE-2011-3544-BD
AVG	Android:RuFraud-I	Android:RuFraud-I	Java:CVE-2011-3544-BD
ESET-NOD32	Android/TrojanSMS.Agent.K	Android/TrojanSMS.Agent.K	a variant of Java/ Exploit.CVE-2011-3544.DF
McAfee	Artemis!9EF6966B98A5	Artemis!BEE5A7C75B6A	RDN/Generic
Avira	ANDROID/SmsAgent.CQ.Gen	ANDROID/SmsAgent.CQ.Gen	EXP/CVE-2011-3544
Sophos	Andr/Jifake-B	Andr/Opfake-A	Mal/Generic-S
Symantec	Android.Fakemini	Android.Fakemini	Trojan.MalJava
IkarusV	Trojan.AndroidOS.FakeInst	Trojan.AndroidOS.FakeInst	Java.CVE
MAX	Malware	malware	Malware
TrendMicro-HouseCall	Suspicious_GEN.F47V0322	AndroidOS_OPFAKE.A,	Suspicious_GEN.F47V0322
Emsisoft	Android.Trojan.FakeInst.CB	Android.Trojan.FakeInst.CB	Gen:Variant.Barys.841
GData	Android.Trojan.FakeInst.CB	Android.Trojan.FakeInst.CB	Gen:Variant.Barys.841
BitDefender	Android.Trojan.FakeInst.CB	Android.Trojan.FakeInst.CB	Gen:Variant.Barys.841
Tencent	Trojan.Android.FakeLogo.aa	Trojan.Android.FakeLogo.aa	Win32.Trojan.Jorik.Hvje
Arcabit	Android.Trojan.FakeInst.CB	Android.Trojan.FakeInst.CB	Trojan.Barys.841

## State-of-the-art

JAR files are collections of individually compressed class files. They are approximately half the size of the original class files^2^. A Java archive (JAR) file encapsulates Java classes and may also contain other resources, such as digital signatures or pictures^12^. JAR files are primarily designed to provide a reliable environment for running small programs embedded in web pages known as applets^12^. The importance of web pages is highlighted, not only for entertainment but also for financial transactions, fulfillment of professional obligations and even for medical appointments. In the expectation of providing protection for web pages, the authorial antivirus specializes in JAR malware.

The modus operandi of commercial antivirus software and Oracle Java (especially Java Security Manager) is used to identify JAR malware with a signature basis. The main problem with this strategy is that for a new virtual plague to be signed, it must be detected that certain computers have been infected. Considering the limitations of commercial antivirus software and Java security managers, the latest technology proposes extracting and analyzing files through a statistical learning machine. Artificial intelligence can automate many tasks by analyzing thousands of files, extracting their functions, and classifying them.

Lima et al. (2021) created an antivirus capable of detecting PE file (Windows) malware with an average accuracy of 98.32%^6^. Lima et al. (2021) used four architectures for each MLP learning function. The first architecture has a single hidden-layer containing 100 neurons. The second architecture has two hidden layers, each one with 100 neurons. The third architecture employs a hidden layer; however, 500 neurons are used. The fourth architecture has four hidden layers, each one with 1261 neurons. Within the fourth architecture, the number of 1261 neurons is because Hecht–Nielsen’s empirical formula is applied. Hecht–Nielsen (1987) noted that the neural network can be configured as a hidden layer with exactly 2n+1 nodes, where n is the number of input nodes. Antiviruses made by Lima et al. (2021) used shallow neural networks.

Deep net-based antiviruses have also achieved excellent accuracy. Vinayakumar et al. (2019) achieved an average accuracy of 98.90% in detecting PE file malware^13^. The deep network structure has 34 layers^14^. The network is trained with 500 epochs with a training batch size of 64 and a learning rate 0.01.

Su et al. (2018) achieved an average accuracy of 94.00% in detecting Internet of Things (IoT) malware^15^. The deep network structure has 6 layers. There are 3 layers with learnable weights: 2 convolutional layers and 1 fully-connected layer. The network is trained with 5,000 iterations with a training batch size of 32 and a learning rate of 0.0001.

The recurrent neural network model has been applied to malware detection from personal computers to the Internet of Things^16,17^. MANIATH, S. *et al.* (2017) created antivirus software to detect ransomware by employing long short-term memory (LSTM) deep networks^16^. LSTM is a recurrent neural network (RNN) architecture used in the field of deep learning. Unlike standard feedforward neural networks, LSTM has feedback connections. In an antivirus software made by Maniath et al. (2017), the training network consists of 3 layers with 64 LSTM nodes in each layer. The deep network is trained for 500 epochs with a batch size of 64. Maniath et al. (2016) achieved an average accuracy of 96.67%.

In addition to antivirus software, LSTM networks have also been employed in firewalls^17^. The goal is to segregate malicious network traffic from benign traffic. A non-intelligent firewall has static formulas that block selected user ports and applications. If the user needs a blocked port for an application, the blockade must be manually disabled, which can result in opening this port for malicious traffic. In a firewall made by Wozniak et al. (2015), the training network consists of 16 layers of LSTM nodes ranging from 256 to 2 neurons in the final layer. The assumption is that the deep network is trained for 1,000 epochs. The firewall made by Wozniak et al. (2015) achieved an average accuracy of 99.99%.

The antivirus made by Hou (2016) detected Android malware by employing a deep belief network^18^ and built on a stack of Restricted Boltzmann Machine (RBM) that constitute the deep belief network, where the trained activations of one RBM are used as the inputs of the next RBM. The network architecture consists of 3 layers with 200 hidden nodes in each layer. Hou et al. (2016) achieved an average accuracy of 96.66%.

The antivirus made by Hardy et al. (2016) detects PE file (Windows) malware by employing stacked autoencoder deep networks^19^. The decoder attempts to map this representation back to the original input. The deep learning model is trained with 3 hidden layers and 100 neurons at each hidden layer. The encoder maps the input to a hidden representation. Hardy et al. (2016) achieved an average accuracy of 96.85%.

Kalash et al. (2018) proposed deep learning employing VGG-16 for malware classification^20^. Kalash et al. (2018) achieved an average accuracy of 98.52%. The network has an image input size of 224 $\times$ 224. VGG-16 is a convolutional neural network that is 16 layers deep. The pretrained network can classify images into 1000 object categories, such as keyboards, mice, pencils, and many animals. The last 1000 fully-connected VGG-16 is replaced by a fully-connected softmax layer (benign, malware). Herein, we replicate the antivirus made by Kalash et al. (2018). Image input is created through a surface plot made by our dynamic feature extraction.

The disadvantage of the deep net is the long training time. As an aggravating factor, deep networks have lower parallel capabilities because these layers are sequential. Therefore, this layer can only be executed after the upper layer has completed its work. In applications that require frequent training (learning) of antivirus software, this fact may be an obstacle because, on average, 8 (eight) new malware types are created every second^21^. In syntheses, there should be no difference in the antivirus software learning time compared with the rate of new malware generation worldwide.

Deep learning performed by Santos et al. (2019) has a training time compatible with applications that often require training. The deep network presents a single convolutional layer, and there is no data backpropagation. Therefore, since the supercomputer has sufficient computing resources (memory), deep learning made by Santos et al. (2019) has major parallel capabilities. The convolution layer uses 30,000 convolution filters simultaneously.

Instead of random convolutional filters, deep learning made by Santos et al. (2019) developed Principal Component Analysis (PCA) filters that extract the main components of the regions of interest related to the data input vector. Altogether, the technique employs 30 thousand convolutional filters. Each filter is converted to a Toeplitz matrix denoted by $W_{detect} \in \mathrm{I\!R} ^{L^{2} \;x\; R^{2}} , with \;L= (J+W-1)$ for an LxL size feature map. W and J refer to filter size and pixels, respectively^22^.

Herein, we replicate the deep net made by Santos et al. (2019) because its training time is compatible with applications that need frequent training as an antivirus. Deep Learning made by Santos et al. (2019) is not aimed at detecting malware, but at optical character recognition^22^. Instead of digital image processing, the input attributes extract malware features.

Due to the excellent results obtained by deep learning techniques, common sense has been created that deep learning can provide the best accuracy in any application type; in fact, this consideration is untrue. Deep neural networks, specifically convolutional networks, are based on linear filter convolution. Although it has a fundamental role in computer applications, filter convolution is limited to applications when a vector flow gradient is formed.

Consider, for example, biomedical images from mammography devices. The images are full of noise that hinders breast lesion recognition^23^. Therefore, convolution of filters is essential to eliminate noise and, therefore, discard small irregularities in the finding corresponding to potential cancer. Convolutional techniques, such as Gaussian filters, are essential to reducing noise in biomedical images^23^.

As a counterexample, consider the repository image shown in Table 3. The features are completely disconnected from each other despite belonging to the same neighborhood. An application suspected of attempting to determine whether Wi-Fi data has no correlation with accessing the victim’s image gallery or browser. Then, when applying the linear convolution of filters in the repository, illustrated in Table 3, accessing the browser, containing the value 0, is treated as noise. The explanation is that its neighborhood has positive values. In synthesis, the suspect application is accused of accessing the victim’s browser, even the extraction of features having audited the inverse. Convolutional techniques suffer a disadvantage when applied to malware pattern recognition.

To prove our theoretical background, the authorial antivirus employs shallow morphological neural networks instead of deep convolutional networks. As expected, the authorial antivirus has accuracy compared to next-generation antiviruses based on both shallow and deep neural networks. Our antivirus can combine high precision with reduced learning time. To avoid unfair comparisons, the feature extraction stage is standardized by monitoring 6,824 behaviors that the suspicious JAR file can perform when executed purposely.

**Table 3:** Example of a statistical repository based on malware detection.

Features		
Check Wi-fi Data	Access the Browser	Access Image Gallery
1	0	1

## Materials and methods

The present paper aims to elaborate the retrieval of JAR files applied to dynamic analysis (REJAFADA), a dataset that allows the classification of files with the JAR extension between benign and malware. REJAFADA consists of 998 malware JAR samples and 998 other benign JAR samples. The REJAFADA dataset, consequently, is suitable for learning endowed with artificial intelligence (AI), considering that the JAR files presented the same amount in the different classes (malware and benign). The goal is that classifiers that are biased toward a particular class do not have their success rates favored.

In relation to virtual viruses, REJAFADA extracted malicious JAR files from VirusShare, which is a repository of malware samples to provide security researchers, incident responders, forensic analysts, and the morbidly curious access to samples of live malicious code^24^. To catalog the 998 JAR malware samples, it was necessary to acquire and analyze, by authorial script, approximately 3 million malware samples from the reports updated daily by VirusShare.

With respect to benign JAR files, the catalog was given from application repositories such as Java2s.com^25^, and findar.com^26^. All of the benign files were audited by VirusTotal. Then, the benign JAR files contained in REJAFADA had their benevolence attested by the main commercial antiviruses of the world. The obtained results corresponding to the analyses of the benign and malware JAR files resulting from the VirusTotal audit are available for consultation at the REJAFADA virtual address^11^. The purpose of creating the REJAFADA dataset is to give the full possibility for the proposed methodology to be replicated by others in future work. Then, REJAFADA is freely available with all their samples, such as benign malware:

- VirusTotal audits;
- Dynamic analysis of Cuckoo Sandbox.

REJAFADA also makes available in its virtual address and its 998 benign JAR files. In addition, our dataset displays the list of all 998 other JAR files, this time, malware. Then, there is the possibility of acquiring all the malware employed by REJAFADA by establishing agreements and submitting to ViruShare’s terms of use^11^. We conclude that our REJAFADA dataset provides transparency and impartiality to the research and demonstrates the veracity of our results. Therefore, it is expected that REJAFADA will serve as a basis for creating new scientific works aiming at next-generation antiviruses.

All experiments were carried out on a cloud supercomputer equipped with 250 GB of RAM, 8 processors and 300 GB of mass storage. Therefore, there are no unfair comparisons, and state-of-the-art antiviruses are trained and tested on the same supercomputer used by the copyrighted antivirus. It is emphasized that the acquisition of a supercomputer was due to replication and comparison with state-of-the-art works. The authorial antivirus requires low processing and storage capacity. It is emphasized that the authorial antivirus can be used on any conventional desktop computer.

### Research involving human participants and/or animals

The authors declare that no human participants were involved in this research.

### Informed consent

This research did not include health care interventions of human participants.

## Proposed methodology

**Figure 1:**
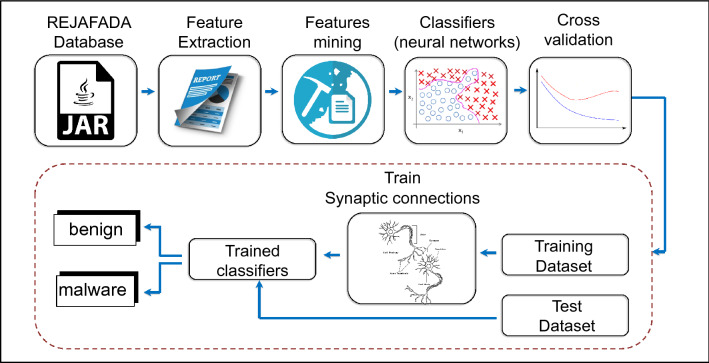
Diagram of the proposed methodology.

Figure 1 shows the diagram of the methodology proposed in the block diagram. Initially, the JAR file, originating from the REJAFADA dataset, is executed to verify the attempt to corrupt the JVM and, in sequence, Windows 7 audited by the Cuckoo Sandbox. The dynamic features are synthesized in Sect. 5.1. Then, the dynamic characteristics of the JAR files are stored in a machine learning repository format.

As a method of feature mining some behaviors audited by the sandbox, are ignored. The adopted mining criterion refers to feature elimination that concerns a single JAR file, for example, process IDs, process names, md5, and sha. After mining features, the relevant behaviors of the JAR samples serve as machine learning input attributes, specifically, artificial neural networks are employed as classifiers. The goal is to group the JAR samples into two classes: benign and malware. The classification stage is explained in detail in Sect. 5.2. Classification results are described in Chapter 6.

### Dynamic feature extraction

The features of JAR files originate through the dynamic analysis of suspicious files. Therefore, in our methodology, the malware is executed to intentionally infect the JVM installed in Window9s 7 audited, in real time (dynamic), by the Cuckoo Sandbox^27^. In total, 6824 features are generated by each JAR file regarding the monitoring of the suspect file in the proposed controlled environment. To facilitate understanding the input layer neurons, our repository extends the description of the attributes audited by the authorial antivirus^11^. Next, the feature groups are detailed:

- Features related to virtual machines. The goal is to verify that the audited file searches to detect whether Bochs, Sandboxie, VirtualBox, VirtualPC, VMware, Xen or Parallels virtual machines are being used through the presence of registry keys (regedit), files, instructions, and device drivers used by them.
- Features related to malware. Checks whether the audited file attempts create Mutexes (single name files, with a function to set a lock/unlock state, which ensures that only one process at a time uses the resources).
- Features related to Bitcoin. Examines whether the tested file attempts to install the OpenCL library, Bitcoins mining tool.
- Features related to bots (machines that perform automatic network tasks, malicious or not, without the knowledge of their owners).
- Features related to browsers. Checks if the file attempted to:
  - modify browser security settings;
  - modify the browser home page;
  - acquire private information from locally installed internet browsers.
- Features related to firewalls. The proposed digital forensics audits whether the file attempts to modify local firewall policies and settings.
- Features related to cloud computing. The file is audited when it attempts to connect to storage services or files from Dropbox, Google, MediaFire, MegaUpload, RapidShare, Cloudflare and WeTransfer.
- Features that seek to disable features of Windows 7 OS and other utilities. The audit checks to determine the file attempts to:
  - modify system policies to prevent the launch of specific applications or executables;
  - disable browser security warnings;
  - disable Windows security features and properties;
- Features associated with network traffic in Windows 7 OS in the PCAP format.
- Features related to Windows 7 OS (Regedit):
  - Changes in associations between file extensions and software installed on the machine;
  - Changes to the current user information;
  - Driver corruption;
  - Changes to the Windows appearance settings and settings made by users, such as wallpaper, screensaver, and themes;
  - Changes to hardware settings.
- Features related to the use of sandboxes. The digital forensics examine whether the file attempted to turn off Windows functions monitored by the Cuckoo Sandbox.
- Features related to ransomware (a type of malware that, by means of encryption, leaves the victim’s files unusable, then requests a redemption in exchange for the normal use of the user’s files; the redemption is usually paid in a nontraceable way, such as bitcoins).
- Features related to exploit-related features that constitute malware attempting to exploit known or unpackaged vulnerabilities, faults or defects in the system or one or more of its components to cause unforeseen instabilities and behavior on both the hardware and software.
- Features related to infostealers, malicious programs that collect confidential information from the affected computer.

In addition to detecting suspicious behaviors, such as API calls, the dynamic analysis also allows reconstituting (cleaning) the operating system (OS) by auditing the malefactions promoted by the malicious file in the OS, assuming that the harm is not statistically irreversible. It should be noted that reconstituting the OS, technically called a vaccine, is important because it is not enough to detect and eliminate the malware to free the victim of its actions. In addition to eliminating malware, it is necessary to undo all of its malfeasances, for instance, having disabled Java security managers from the JVM. Then, if no auditing is provided by the dynamic analysis, it will fall to the cyber watcher to manually monitor any OS changes that will change the slow and stressful process.

### Classifiers

Our antivirus employs artificial neural networks as classifiers. To choose the best setting of the neural network architecture, we employ different learning functions and initial configurations to require a larger number of calculations, such as multiplying the number of neurons in the intermediary layer. The neural network architectures have an input layer containing many neurons regarding the vector of extracted features from the JAR file monitoring in a controlled environment. Therefore, the employed classifiers must have an input layer containing 6,824 neurons. They concern the features of auditing the JAR file. The output layer has two neurons, corresponding to benign and malware samples.

The proposed work resulted in an antivirus composed of extreme learning machines (ELMs) neural networks to detect malware preventively. ELMs are powerful and flexible kernel-based learning machines whose main characteristics are rapid training and robust classification performance^28^. The ELM network is a single hidden-layer network, not recurrent, based on an analytical method to estimate the network output weights in any random initialization of input weights.

ELMs have been widely applied in several areas, such as biomedical engineering^23,29–34^. ELM networks can greatly contribute to advancing the digital security of devices. The proposed paper applies ELMs in the area of information security specifically in the recognition of malware patterns.

Mathematically, in the ELM neural network, the input attributes $x_{it}$ correspond to the set $$\begin{Bmatrix} x_{it} \in \mathrm{I\!R}; \, i=1,\ldots , n; \, t=1,\ldots , \sigma \end{Bmatrix}$$. Therefore, there are *n* extracted features (input neurons) from the application and $\sigma$ training data vectors. The hidden layer $h_{j}$, consisting of *m* neurons, is represented by the set $$\begin{Bmatrix} h_{j} \in \mathrm{I\!R}; \, j \in \mathrm{} N^{*}; \, j=1,\ldots , m \end{Bmatrix}$$. The ELM training process is fast because it is composed of only a few steps. Initially, the input weights $w_{ji}$ and bias $bias \, b_{jt}$ is defined in a random generation. Given an activation function f:R$\rightarrow$R the learning process is divided into three steps:

- Random generation of weight $w_{ij}$, corresponding to the weights between the input and the hidden layers, and bias ${bias \, b_{jt}}$.
- Calculate the matrix H, which corresponds to the output of the hidden-layer neurons.
- Calculate the matrix of the output weights $\,=H \dagger Y$, where $H \dagger$ is the generalized Moore-Penrose inverse matrix of the matrix H, and Y corresponds to the matrix of desired outputs s.

The concept of an inverse matrix is related to the identity matrix. An original square matrix *H* multiplied by its inverse $H^{-1}$ is equal to the identity matrix $H.H^{-1} = I$. However, in cases of a rectangular dimensional matrix, therefore non-square, an approximately inverse matrix $H \dagger$ is generated. This approximately inverse matrix is responsible for polarizing the synaptic weights between neurons. The pseudo-inverse matrix repels the synaptic weights from the decision boundary toward the extremes (poles) of the secondary diagonal.

In mathematical terms, the pseudo-inverse matrix $H \dagger$ uses the singular value decomposition $H = U \Sigma V^*$, where *U* is a real or complex $n\times n$ unit matrix and *n* is the total input neuron. $\Sigma$ is a diagonal rectangular $n \times \sigma$ matrix with real non-negative numbers on the main diagonal, *n* is the total input neuron and $\sigma$ is the total of training data vectors. V* (the transposed conjugate of V) is a real or complex $\sigma \times \sigma$ unitary matrix. The diagonal entries $\Sigma _{i,t}$ of $\Sigma$ are named singular values of *H*. The *n* columns of U and the $\sigma$ columns of V are the named singular left vectors and singular right vectors of *H*, respectively. The pseudo-inverse of *H* is then equal to $H \dagger = V \Sigma ^{-1} U^*$.

The output of the hidden-layer neurons, corresponding to the matrix H, is computed by the kernel *K*, dataset inputs and weights between the input and the hidden layers shown in Eq. (1).

1 $$\begin{aligned} H_{jt} = \begin{bmatrix} K(1,1) &{} K(1,2) &{} \cdots &{} K(1,n) \\ K(2,1) &{} K(2,2) &{} \cdots &{} K(2,n) \\ \vdots &{} \vdots &{} \ddots &{} \vdots \\ K(\sigma ,1) &{} K(\sigma ,2) &{} \cdots &{} K(\sigma ,n) \end{bmatrix} \end{aligned}$$

Instead of using conventional kernels, authorial kernels are used for ELMs. We employ mELMs (morphological ELMs), ELMs with hidden-layer cores based on the morphological operators of erosion and dilation image processing. Kernels are mathematical functions employed as a method for learning neural networks. This learning method enables the creation of nonlinear data mapping. Thus, there is no need to increase the number of adjustable parameters, as in the learning rate used in networks with backward propagation. There are two fundamental morphological operations, erosion and dilation. The theory of mathematical morphology can be considered constructive since all operations are built based on erosion and dilation. Mathematically, erosion and dilation are characterized according to Eq. (2) and Eq. (3), respectively:

2 $$\begin{aligned} \varepsilon _{g} (\textit{f}) (\textit{u})= & {} \underset{v \in S}{\bigcap }f (\textit{v}) \, \vee \, {\overline{g}} (\textit{u - v}) \end{aligned}$$

3 $$\begin{aligned} \varepsilon _{g} (\textit{f}) (\textit{u})= & {} \underset{v \in S}{\bigcup }f (\textit{v}) \, \wedge \, g (\textit{u - v}) \end{aligned}$$

Where $f:S\rightarrow [0,1]$ and $g:S\rightarrow [0,1]$ are normalized images in the form of a matrix named *S*, where $S\in \mathrm{I\!N}^2$ format. Pixel is defined by the Cartesian pair (*u*, *f*(*u*)), where *u* is the position related to value *f*(*u*). *v* is the matrix of *f*(*u*), encompassed by *g*. The operators are associated with the maximum operation, while and are associated with the minimum operation. g is the structuring element for both erosion and dilation^8^. ${{\overline{g}}}$ is the negation of *g*.

In Eq. (2) initially negates the structuring element ${{\overline{g}}}$. Then, the operation of maximum $\vee$ denoted by $f(v) \vee {{\overline{g}}}(u-v)$, where *f*(*v*) refers to the original image matrix *f* covered (matched) by ${{\overline{g}}}$. *f*(*v*) is technically named the active region of the image. Finally, the value $\epsilon _g (f)(u)$, in position *u*, of the eroded image receives the minimum value between the maximums via the $\cap$ operator. $\epsilon _g (f)(u)$ obtains the value 0 associated with absolute black. Erosion overlays ${{\overline{g}}}$ to the original image *f*. The goal is that areas similar to ${{\overline{g}}}$ expand^8^. By associating the 1s with absolute white and the 0s with absolute black, erosion enhances darker areas and eliminates regions with high intensity^8^.

Eq. (3) shows the performance of the morphological dilation operation. Due to mathematical precedence, the minimum $\wedge$ operation denoted by $f(v)\wedge g(u-v)$, occurs, where *f*(*v*) refers to the original image matrix *f* covered (matched) by *g*. Therefore, the $\delta _g (f)(u)$ at the *u* position of the expanded image receives the maximum value between the minimums through the $\cup$ operator. Dilation superimposes the structuring element *g* on the original image *f*. The goal is that areas similar to *g* expand. By associating 1’s with absolute white and 0’s with absolute black, the dilation increases the areas with more intense tonality and eliminates the dark regions^8^.

Our antivirus utilizes mELMs. They are inspired by mathematical morphology based on nonlinear erosion and dilation operators. As indicated by Eq. (2) concerning the erosion image operator, the erosion ELM kernel can be defined together with Eq. (4), where $$\begin{Bmatrix}i\in \mathrm{I\!N}^*,i=1,\ldots , n\end{Bmatrix}$$,$$\begin{Bmatrix}j\in \mathrm{I\!N}^*, j=1,\ldots , m\end{Bmatrix}$$, $$\begin{Bmatrix}t\in \mathrm{I\!N}^*,t=1,\ldots , \sigma \end{Bmatrix}$$. There are *n* neurons in the entry layer (without bias), *m* neurons in the hidden layer, and *v* training data vectors.

4 $$\begin{aligned} K_{\epsilon } (\textit{t,i}) = (x_{it} \vee {{\overline{w}}}_{ji}) + b_{jt} \end{aligned}$$

Similar to the erosion kernel, Eq. (5) defines the dilation kernel inspired by Eq. (3) and refers to the morphological operator of dilation.

5 $$\begin{aligned} K_{\delta } (\textit{t,i}) = (x_{it} \wedge w_{ji}) + b_{jt} \end{aligned}$$

**Figure 2:**
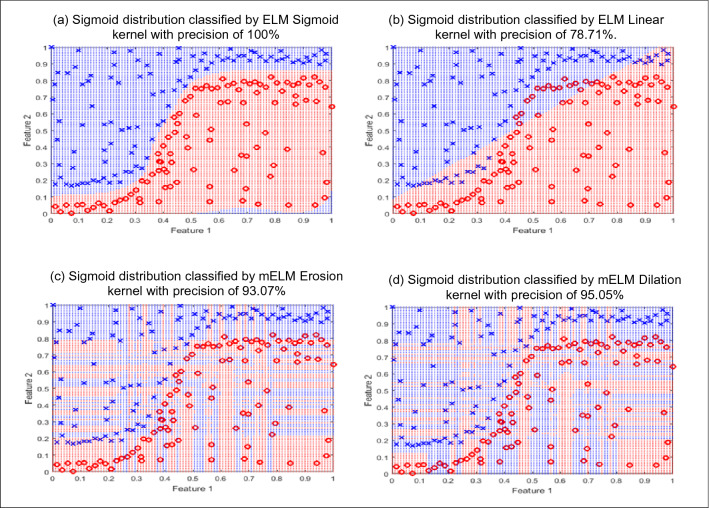
(**a**) Successful performance of the kernel compatible with dataset. (**b**) Inaccurate classification of the Linear kernel in a non-linearly separable distribution. (**c**,**d**) Successful performances by Dilation and Erosion kernels.

To achieve good performance in ELMs, it is necessary to choose a kernel that can optimize the decision boundary for the presented problem, as seen in Fig. 2a. A linear kernel obtains great results when used to solve a linearly separable problem. However, when used to solve nonlinearly separable problems, as shown in Fig. 2b, for a sigmoid distribution, it does not perform satisfactorily. A great generalization capability of the neural network may depend on a fine-tuned kernel choice. The best kernel may be subordinate to the problem to be resolved.

As a side impact, investigating different kernels is a stressful affair that involves cross validation combined with different random initial conditions. However, the investigation of distinct kernels may be necessary; otherwise, the neural network is composed of a mismatched kernel to generate unsatisfactory results.

Figure 2c,d show the performance of the mELM kernel erosion and dilation, with respective accuracies of 93.07% and 95.05%. It is possible to notice when analyzing the figures that the mELMs have the capacity to accurately map the different distributions referring to different problems.

The effectiveness of our morphological neural networks is due to their ability to adapt to any type of distribution since their mapping does not obey any conventional geometric figure. The mapping of decision borders is made by their training data, the very position in *n*-dimensional space that will determine whether that surrounding region belongs to class 1 or class 2. *n* represents the number of neurons in the input layer. Therefore, our mELM kernel can naturally detect and model the *n*-dimensional regions divided into different classes by using Mathematical Morphology.

To prove our theoretical background, the authorial antivirus employs shallow morphological neural networks instead of deep convolutional networks. We claim that it is not computational complexity that will make the neural network more efficient. Adequacy to the target problem makes the neural network efficient regardless of the number of calculations. A shallow linear network, for example, can solve a linearly separable problem as well as a state-of-the-art deep learning network that consumes days to complete its training.

The authorial morphological kernels present an important relationship with the created repository. The justification is that mathematical morphology can detect and segment the boundaries of target objects, preserving the relationships of bodies through the use of the mathematical theory of intersection, union, and difference of sets^8^. When considering the example contained in Table 3, authorial morphological kernels can process fully segregated regions, preserving their boundaries. By region, we denote an area containing continuously congruent values.

## Results of ELM networks

We employ seven different kernel types for the ELM neural networks. In the state of the art, three of these kernels were described by Huang et al. (2012): wavelet transform, hard limit and tribas (triangular base function)^28^. In addition, authorial kernels are employed: fuzzy-dilation, fuzzy-erosion, dilation and erosion.

fmELMs have been successful in treating biomedical images, specifically in detecting and classifying breast cancer^32,33^. The fmELMs constitute linear functions, inspired by mathematical morphology, with optimized learning time compared to the mELMs. Despite the low learning time, fmELMs are not completely suitable for nonlinear distributions such as sigmoid and sinusoidal distributions.

The wavelet kernel has no hidden layer. The calculations are based on transforming the input data and can work similarly to kernels containing architectures with hidden layers^28^. A good generalization capability of these channels depends on an adjusted choice of parameters $(C,\gamma )$^28^. The cost parameter C refers to a reasonable equilibrium point between the hyperplane margin width and the classification error minimization in relation to the training set. The kernel parameter $\gamma$ controls the decision boundary as a function of classes^28^. There is no universal method in the sense of choosing the parameters $(C,\gamma )$.

In the present work, there is an investigation of the parameters $(C,\gamma )$ inspired by the method proposed by HUANG *et al.* (2012), which consists of training increasing sequences of C and $\gamma$, mathematically, $2^n$, where $$n=\begin{Bmatrix}-24,-10,0,10,25\end{Bmatrix}$$^28^. The hypothesis is to verify whether these parameters with values different from the standards $(C=1,\gamma =1)$ generate better results.

Each combination employs cross validation through the k-fold method, where k=10. The goal is that the results achieved are not influenced by the training and test sets. For this, the total number of samples is divided into ten parts. In the first iteration, the first part is the test set, while the others are reserved for training. This rotation occurs for ten cycles until all ten parts have been applied to the test phase. The ELM accuracy is the arithmetic mean of the hit rate obtained in the ten cycles. As previously mentioned, in the ELM network, there is no data backpropagation. Therefore, the objective of the k-fold cross-validation method is not to establish a stopping criterion to avoid overfitting (excess training) but to verify that the classifier undergoes abrupt changes in its accuracy depending on the training and testing sets. Moreover, methodological care should be taken to randomly select benign and malware samples for each fold. The objective is that biased classifiers, in relation to a given class, do not have their accuracy rates favored.

Table 4 details the results obtained by the ELM neural networks with a wavelet kernel. Each row in this table contains 10 executions referent to cross validation of the k-fold method, where k=10. In relation to precision in the test phase, the maximum average performance is 56.01% in the distinction between benign and malware cases with the parameters $(C, \gamma ) = (2^{-24}, 2^{0})$. In Table 4, there are only the best and worst-case descriptions, in this order, for each ELM kernel.

The hard limit, tribas (triangular basis function),fuzzy-dilation, and fuzzy-erosion, dilation and erosion networks employ hidden-layer architectures. At this point, there is an investigation regarding the number of neurons in the hidden layer of these kernels. The hypothesis is to verify whether architectures that require a higher volume of calculations, such as multiplying the number of neurons in the hidden layer, can produce better accuracy rates compared to architectures that demand fewer calculations. Two architectures are evaluated; they employ 100 and 500 neurons in their respective hidden layers. These architectures have a background of excellent accuracy in the application of ELM networks in the area of biomedical engineering^6^.

Table 5 details the results obtained by the ELM neural networks with the hard limit, tribas (triangular basis function), fuzzy-dilation, and fuzzy-erosion, dilation and erosion kernels. Each row in Table 5 contains 10 distinct executions referring to the k-fold method, where k=10. Regarding precision, the maximum average performance is 91.58% with a standard deviation of 1.77% through the dilation kernel endowed with 500 neurons in its hidden layer.

**Table 4:** Result of ELM networks. The $(C, \gamma )$ parameters vary according to the set $\{2^{-24}, 2^{-10}, 2^{0}, 2^{10}, 2^{25}\}$. There are only the best and worst-case descriptions.

Kernel	(C, $\gamma$)	Train rate (%)	Test rate (%)	Train time (sec.)	Test time (sec.)
Wavelets	$(2^{-24}, 2^{0})$	**100.00** ± **0.00**	**56.01** ± **2.78**	3.11 ± 0.07	0.76 ± 0.03
	$(2^{10}, 2^{25})$	67.40 ± 1.97	47.91 ± 3.76	3.12 ± 0.08	0.78 ± 0.03

**Table 5:** Result of ELM Networks. The number of neurons in the hidden layer varies according to 100, 500.

Kernel	Neurons	Train rate (%)	Test rate (%)	Train time (sec.)	Test time (sec.)
Hard limit	100	50.03 ± 0.00	49.75 ± 0.00	0.48 ± 0.01	0.03 ± 0.01
	500	50.03 ± 0.00	49.75 ± 0.00	2.51 ± 0.03	0.13 ± 0.02
Tribas	100	50.00 ± 0.03	50.00 ± 0.26	0.49 ± 0.02	0.02 ± 0.01
	500	50.00 ± 0.03	50.00 ± 0.26	1.76 ± 0.05	0.14 ± 0.01
Fuzzy-Dilation	500	95.71 ± 0.28	87.28 ± 2.40	2.01 ± 0.05	0.14 ± 0.02
	100	84.37 ± 0.29	81.61 ± 2.32	0.55 ± 0.02	0.03 ± 0.01
Fuzzy-Erosion	500	95.70 ± 0.33	87.72 ± 2.55	2.16 ± 0.07	0.16 ± 0.01
	100	84.67 ± 0.37	82.16 ± 1.93	0.65 ± 0.01	0.03 ± 0.01
Dilation	500	**97.63** ± **0.13**	**91.58** ± **1.77**	52.36 ± 0.57	5.68 ± 0.05
	100	81.20 ± 0.35	78.86 ± 1.18	7.34 ± 0.18	0.77 ± 0.02
Erosion	500	78.38 ± 0.24	70.58 ± 2.81	53.23 ± 2.41	5.78 ± 0.23
	100	54.99 ± 0.11	53.05 ± 1.95	8.17 ± 0.15	0.86 ± 0.03

Significant values in bold.

## Results in relation to the state-of-the-art

In this section, authorial antiviruses are compared with state-of-the-art antiviruses. To avoid unfair comparisons, the feature extraction stage is standardized by monitoring 6,824 behaviors that the suspicious JAR file can perform when executed purposely. Authorial antiviruses employ shallow morphological neural networks. Our antivirus is endowed with an mELM dilation kernel and contains 500 neurons in its hidden layer.

In contrast, the antivirus made by Lima et al. (2021) employs shallow neural networks based on backpropagation. Lima et al. (2021) investigated eleven distinct learning functions to optimize the accuracy of their antiviruses. For each learning function, Lima et al. (2021) explored 4 hidden-layer architectures.

Our authorial antivirus is also compared to antiviruses based on deep neural networks. In the present work, the antiviruses made by Su et al. (2018), Vinayakumar et al. (2019), Maniath et al. (2017), Wozniak et al. (2015), Hou et al. (2016), Hardy et al. (2016), and Kalash et al. (2018) are replicated. We replicated these deep learning works employing our dataset.

Best accuracy was achieved by the antivirus made by Vinayakumar et al. (2019). This deep network structure containing 34 sequential convolutional layers^14^. The network made by Vinayakumar et al. (2019) is trained with 500 epochs with a training batch size of 64 and learning rate of 0.01. As a side effect, in our experiments, the antivirus made by Vinayakumar et al. (2019) consumes about two (2) days in order to complete its training in a supercomputer.

Finally, the authorial antivirus is also compared to deep learning made by Santos et al. (2019). This state-of-the-art work does not aim at malware detection but at optical character recognition. Therefore, the deep net made by Santos et al. (2019) undergoes adaptation in its input neuron layer. Instead of digital image processing, the input attributes concern the extraction of malware features. The deep learning method developed by Santos et al. (2019) presents a single convolutional layer, and there is no data backpropagation. The convolutional layer employs all of its 30,000 filters simultaneously so that the training time is compatible with applications that need training frequently as antiviruses.

**Figure 3:**
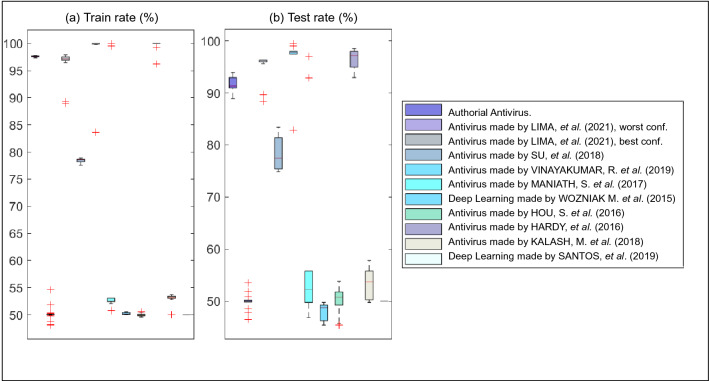
Boxplots referring to the accuracies of the authorial antivirus and the state-of-the-art.

**Figure 4:**
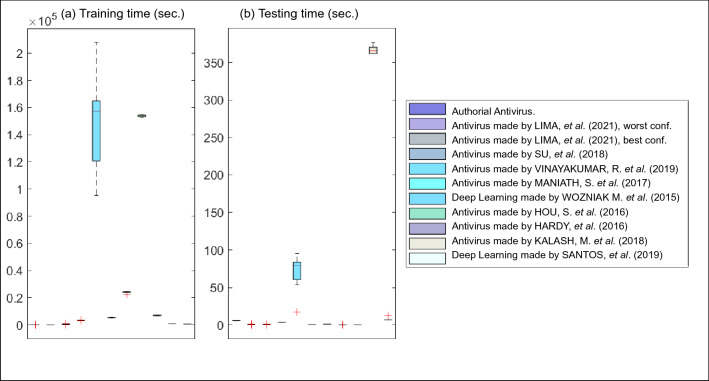
Boxplots regarding the processing times of the authorial antivirus and the state-of-the-art.

Figures 3 and 4 are graphical representations of the results described in Table 6. Figure 3a presents the boxplots from the training stage in relation to authorial antivirus and state-of-the-art methods. The best average accuracy, resulting from the training, is 98.33% through antiviruses made by Vinayakumar et al. (2019). Deep learning made by Santos et al. (2019) obtains a training accuracy of 50% on average. The antivirus made by Lima et al. (2021) obtained average accuracies of 50.26% and 96.71% in its worst and best scenarios, respectively. These results are obtained using the learning functions “batch training-learning rules” and “conjugate gradient backpropagation with Fletcher-Reeves updates”, respectively, with 100 neurons in their hidden layers. The authorial antivirus obtains an average performance of 97.63% with a standard deviation of 0.13%. Therefore, our antivirus has the advantage of not suffering abrupt changes due to the initial conditions (synaptic connections and cross validation).

Figure 3b shows the boxplots for the best accuracy in the test phase. The best average accuracy, resulting from the test, is 96.54% through the antivirus made by Vinayakumar et al. (2019). Deep learning made by Santos et al. (2019) achieves an average performance of 50%. The authorial antivirus obtains an average accuracy of 91.58%. The antivirus made by Lima et al. (2021) achieves a mean accuracy of 50.26% and 95.67% in its worst and best scenarios, respectively. Therefore, it is corroborated that neural networks based on backpropagation can suffer major variations in their accuracies, depending on their configuration parameters. Then, the decision made by Lima et al. (2021) is satisfactory. This state-of-the-art antivirus explores different learning functions, gradients and architectures to optimize the accuracy of its neural networks based on data backpropagation. It should be emphasized that without investigating the parameters of backpropagation-based networks, there is no guarantee that the antivirus made by LIMA *et al.* (2021) will always operate in its best configuration.

A probable reason for the failure of most deep learning-based antiviruses concerns the use of a repository created from dynamic analysis. Originally, state-of-the-art antiviruses employ static analysis when malware is usually converted into an image to serve as an input attribute for deep networks. This solution allows the appearance of a vector gradient since the applications have a specific predefined structure. In contrast, herein, we apply dynamic analysis. The chain of events invoked by the suspect applications may not follow a gradient, as illustrated in Table 3. Therefore, deep neural networks based on linear filter convolution can work weakly despite the large volume of computations.

Figure 4a,b present the boxplots referring to the times spent during the training and test phases, respectively. Authorial antivirus consumes only 52.36 seconds on average to conclude its training. In relation to training time, the antivirus made by Vinayakumar et al. (2019) is slower since it uses a deep network recurrent structure. The antiviruses made by Vinayakumar et al. (2019) and Hou et al. (2018) consume approximately two (2) days, each one, to complete their training. The deep learning made by Santos et al. (2019) consumes 695.26 seconds in its training since 30,000 convolutional filters are employed in parallel, and there is no data backpropagation. The antivirus made by Lima et al. (2021) concludes its training on the order of seconds because it employs shallow neural networks. Regarding the time consumed during the test phase, all techniques consumed very close times without great discrepancies.

Table 7 shows the confusion matrices of the techniques presented in Table 6 in percentage terms. The confusion matrix is important to verify the quality of supervised learning. In Table 7, B. and M. are abbreviations of benign and malware. The desired classes are arranged on the vertical label, while the obtained classes are arranged on the horizontal label. On a confusion matrix, the main diagonal is occupied by cases whenever the obtained class coincides with the expected class, named true positive cases. Then, a good classifier has the main diagonal occupied by high values, and other elements have low values. Table 7 shows the main diagonals emphasized in bold. Our antivirus, in the test phase, mistakenly classified on average 10.79% of cases as benign when they were malware cases (false negative). Similarly, there is a mean classification of 5.49% of cases said to be malware when they are benign applications (false-positive).

Regarding Table 7, sensitivity and specificity refer to the ability of the antivirus to identify malware and benign applications, respectively. The proposed work presents the confusion matrix in percentage terms to facilitate the interpretation of sensitivity and specificity. In synthesis, the sensitivity and specificity are presented in the confusion matrix, described in Table 7. For example, the proposed antivirus averages 94.51% with respect to both sensitivity and true positives. Following the same reasoning, authorial antivirus obtains, on average, 89.21% for both specificity and true negatives.

Table 8 shows the parametric t-students and nonparametric Wilcoxon hypothesis tests between our antivirus and the state-of-the-art. It is possible to conclude that our authorial antivirus is significantly different in comparison to all other samples. The explanation is that in both the parametric t-students and the nonparametric Wilcoxon tests, the null hypothesis is rejected. Therefore, the samples are statistically distinct.

Authorial antiviruses demonstrate a major advantage when compared to the state-of-the-art methods. Our antivirus achieves an average performance of 91.58% within an average training time of 52.36 seconds. This relationship between percentage accuracy and training time in reverse order is broadly employed in biomedical engineering^30^. It is admitted that establishing this relationship assumes an important function in information security since 8 (eight) new malware types are released every second^21^. Therefore, paradoxically, a newly launched antivirus may already be obsolete and require new training through a newly discovered vulnerability. In syntheses, the learning time of an antivirus should not be discrepant in comparison to the rate of new malware creation worldwide.

**Table 6:** Comparison among the authorial antivirus and the state-of-the-art.

Technique	Train rate (%)	Test rate (%)	Train time (sec.)	Test time (sec.)
Authorial Antivirus	97.63 ± 0.13	91.58 ± 1.77	52.36 ± 0.57	5.68 ± 0.05
Antivirus made by Lima et al. (2021), worst c.^6^	50.26 ± 0.89	50.26 ± 0.71	9.67 ± 1.76	0.44 ± 0.14
Antivirus made by Lima et al. (2021), best c.^6^	96.71 ± 2.10	95.67 ± 1.85	580.07 ± 228.44	0.46 ± 0.19
Antivirus made by Su et al.^15^	78.37 ± 0.47	78.31 ± 3.37	3337.15 ± 19.08	3.53 ± 0.14
Antivirus made by Vinayakumar et al.^13^	**98.33** ± **5.16**	**96.54** ± **4.84**	149968.09 ± 33112.72	71.64 ± 23.13
Antivirus made by Maniath et al.^16^	61.80 ± 20.01	60.01 ± 18.61	5483.06 ± 161.34	0.27 ± 0.01
Deep Learning made by Wozniak et al.^17^	50.22 ± 0.19	48.05 ± 1.65	24102.06 ± 629.56	1.14 ± 0.02
Antivirus made by Hou et al.^18^	50.00 ± 0.30	50.01 ± 2.63	153916.96 ± 725.12	0.08 ± 0.01
Antivirus made by Hardy et al.^19^	99.57 ± 1.17	96.49 ± 1.89	6854.74 ± 300.59	0.14 ± 0.01
Antivirus made by Kalash et al.^20^	52.93 ± 1.06	53.40 ± 2.84	897.77 ± 8.32	366.45 ± 4.99
Deep Learning made by Santos et al.^22^	50.00 ± 0.00	50.00 ± 0.00	695.26 ± 29.67	7.90 ± 2.56

Significant values in bold.

**Table 7:** Confusion matrix of the authorial antivirus and the state-of-the-art (%).

Technique		Train		Test	
		M.	B.	M.	B.
Authorial Antivirus	M.	**99.06** ± **0.25**	0.94 ± 0.25	**94.51** ± **1.83**	5.49 ± 1.83
	B.	3.72 ± 0.16	**96.28** ± **0.16**	10.79 ± 3.25	**89.21** ± **3.25**
Antivirus made by Lima et al. (2021),	M.	**42.76** ± **49.24**	57.24 ± 49.24	**42.56** ±** 49.07**	57.44 ± 49.07
worst conf.^6^	B.	42.60 ± 49.51	**57.40** ± **49.51**	42.64 ± 49.45	**57.36** ± **49.45**
Antivirus made by Lima et al. (2021),	M.	**94.27** ± **2.62**	5.73 ± 2.62	**93.42** ± **1.99**	6.58 ± 1.99
best conf.^6^	B.	0.84 ± 1.65	**99.16** ± **1.65**	2.06 ± 1.77	**97.94** ± **1.77**
Antivirus made by Su	M.	**74.86** ± **0.65**	25.14 ± 0.65	**74.86** ± **4.09**	25.14 ± 4.09
*et al.*^15^	B.	16.86 ± 1.89	**83.14** ± **1.89**	16.86 ± 4.49	**83.14** ± **4.49**
Antivirus made by Vinayakumar	M.	**97.63** ± **7.39**	2.37 ± 7.39	**96.60** ± **6.96**	3.40 ± 6.96
et al.^13^	B.	0.46 ± 1.33	**99.54** ± **1.33**	3.12 ± 2.11	**96.88** ± **2.11**
Antivirus made by MANIATH, S	M.	**95.02** ± **15.67**	4.98 ± 15.67	**88.54 **± **15.99**	11.46 ± 15.99
et al.^16^	B.	34.14 ± 23.38	**65.86** ± **23.38**	35.52 ± 22.88	**64.48** ± **22.88**
Deep Learning made by WOZNIAK, M.	M.	**70.00** ± **48.30**	30.00 ± 48.30	**70.00 **± **48.30**	30.00 ± 48.30
et al.^17^	B.	70.00 ± 48.30	**30.00** ± **48.30**	70.00 ± 48.30	**30.00** ± **48.30**
Antivirus made by HOU, S.	M.	**100.00** ± **0.00**	0.00 ± 0.00	**100.00** ± **0.00**	0.00 ± 0.00
et al.^18^	B.	0.00 ± 0.00	**100.00** ± **0.00**	0.00 ± 0.00	**100.00** ± **0.00**
Antivirus made by HARDY,	M.	**99.92** ± **0.22**	0.08 ± 0.22	**98.01** ± **2.03**	1.99 ± 2.03
et al.^19^	B.	0.75 ± 2.02	**99.25** ± **2.02**	4.90 ± 2.30	**95.10** ± **2.30**
Antivirus made by KALASH, M.	M.	**56.18 **± **2.25**	43.82 ± 2.25	**57.39** ± **5.97**	42.61 ± 5.97
et al.^20^	B.	43.08 ± 15.14	**46.92 ± 16.49**	42.77 ± 15.12	**47.23** ± **16.68**
Deep Learning made by SANTOS,	M.	**100.00** ± **0.00**	0.00 ± 0.00	**100.00** ± **0.00**	0.00 ± 0.00
et al.^22^	B.	100.00 ± 0.00	**0.00** ± **0.00**	100.00 ± 0.00	**0.00** ± **0.00**

**Table 8:** T-students and Wilcoxon hypothesis test of the authorial antivirus and the state-of-the-art.

Comparison	t-students (parametric test)		Wilcoxon (non-parametric test)	
	Hypothesis	*p*-value	Hypothesis.	*p*-value
Authorial Antivirus *vs* Antivirus made by Lima et al. (2021), worst conf.	1	4.2134e−41	1	1.30487e−11
Authorial Antivirus *vs* Antivirus made by Lima et al. (2021), best conf.	1	3.81625e−09	1	2.86398e−09
Authorial Antivirus *vs* Antivirus made by Su et al. (2018)	1	6.621e−19	1	2.5046e−11
Authorial Antivirus *vs* Antivirus made by Vinayakumar et al. (2019)	1	3.803e−06	1	8.83703e−08
Authorial Antivirus *vs* Antivirus made by Maniath et al. (2017)	1	7.16622e−11	1	1.04946e−05
Authorial Antivirus *vs* Deep Learning made by Wozniak et al. (2015)	1	9.26937e−41	1	2.14306e−11
Authorial Antivirus *vs* Antivirus made by Hou et al. (2016)	1	2.98566e−36	1	2.37833e−11
Authorial Antivirus *vs* Antivirus made by Hardy et al. (2016)	1	1.33009e−11	1	5.13671e−10
Authorial Antivirus *vs* Antivirus made by Kalash et al. (2018)	1	1.68447e−35	1	2.5046e−11
Authorial Antivirus *vs* Deep Learning made by Santos et al. (2019)	1	5.42386e−42	1	1.02645e−12

## Conclusion

Each year, thousands of malware types are developed in growing and continuous proportions^5^. Therefore, it is of vital importance that malware detection platforms provide preventative cyber surveillance mechanisms that meet customer demands. Otherwise, in scenarios where there is a failure to identify the malicious application, sensitive customer data are likely to be made available to unauthorized persons. Worldwide, vulnerabilities in Java account for 91% of all monitored cyber infections^4^. In addition to personal computers, Java exploits can corrupt corporate web applications and are responsible for most web-based threats^4^.

Thus, it is inferred that selecting the antivirus has an important role in combating virtual plagues. In our evaluation, the variation in JAR malware detection ranged from 0% to 99.10%, depending on which commercial antivirus was chosen. The present paper analyzed 86 commercially available antiviruses. In this paper, the VirusTotal platform was used to automatically submit malware to the antiviruses. It should be emphasized that in VirusTotal, there is not the possibility of choosing the shareware version of antiviruses. Then, it was not possible to perform comparisons among the free and commercial resources of the same antivirus. It is deduced that the services offered in the shareware versions perform significantly inferior to the full versions.

On average, 31.39% of the paid antivirus (full) submitted to our evaluation, and they could not detect any of the JAR malware. The flaw in the effectiveness of commercial antiviruses concerning large-scale, real-time services was verified. It is noteworthy that in our study, the JAR malware analyzed are in the public domain, employed in malicious activities, and with their performances classified by incident responders. Even so, more than one-third of the evaluated commercial antiviruses had no knowledge about the existence of JAR malware.

To supply the limitations of commercial antiviruses, artificial intelligence-based antiviruses can audit thousands of malware types and learn, statistically their malicious characteristics. Therefore, after learning, intelligent antiviruses can identify and classify newly created malware according to the comparison between their features and those cataloged during their learning phase. Hence, there would be no need to wait for a client to be contaminated and subsequently report a suspicious attack as if it were at that moment, the antivirus takes some action with respect to the discovery of new malware. Intelligent antiviruses enable preventive detection of virtual threats in a controlled environment before they reach customers’ machines.

We created an antivirus capable of classifying JAR files between benign and malware. Overall, our antivirus statistically monitors and evaluates 6,824 actions that the suspected JAR file can perform when executed in JVM contained in Windows 7. In a controlled environment, our antivirus monitors articles in the registry (database) of the OS, traces of calls performed by all processes spawned by the malware, files being created, deleted and downloaded by the malware during its execution, memory dumps of the malware processes, and network traffic trace. Pattern recognition, regarding the 6,824 suspicious actions, is performed by ELM.

Instead of conventional kernels, authorial kernels are employed for ELMs. The ELM network has as its main characteristic the training speed and data prediction when compared to conventional neural networks. In this work, we employed the morphological ELM (mELM) neural network, an ELM with a hidden-layer kernel, which is inspired by erosion and dilation image processing morphological operators. The authorial dilation kernel can distinguish Java malware from benign applications in 91.58% of cases, accompanied by a training time of 52.36 seconds.

The explanation of the success of our morphological learning machines concerns their capacity to model any borderline decision since their mapping does not comply with ordinary geometric surfaces such as ellipses and hyperboles employed by classic neural network systems. Borderline decision mapping, performed by our morphological kernels, uses the values of samples reserved for training. Our morphological learning machine interprets the boundary decision of the neural network as an *n*-dimensional image, where *n* is the number of extracted features, including different shapes that can be described by using mathematical morphology. Therefore, our morphological machine kernels naturally handle the delineation and modeling of regions mapped to different classes of any machine learning repository.

Our antivirus can be extended to provide cyber protection to other operating systems equipped with JVM. Then, the intention is to extend our antivirus to other operating systems in addition to Windows. The future goal is to apply our methodology to the Android system since smartphones and tablets are gradually becoming indispensable in contemporary society. In addition, the introduction of the Internet of Medical Things (IoMT) has assisted researchers from both the IT industry and the health care sector in advancing medical treatment^35,36^. Additionally, as a future objective, our antivirus aims to audit OSs equipped with JVM specializing in financial transactions such as smart credit cards, smart transport passes and lottery terminals.
